# Renal Cell Carcinoma of the Kidney with Synchronous Ipsilateral Transitional Cell Carcinoma of the Renal Pelvis

**DOI:** 10.1155/2013/194127

**Published:** 2013-06-13

**Authors:** Dogan Atilgan, Nihat Uluocak, Bekir Suha Parlaktas

**Affiliations:** Gaziosmanpasa University Faculty of Medicine Department of Urology, 60100 Tokat, Turkey

## Abstract

A 73-year-old man was admitted to our clinic with flank pain and gross macroscopic hematuria. Radiologic examination revealed a solid mass in the left kidney and additionally another mass in the ureteropelvic junction of the same kidney with severe hydronephrosis. Left nephroureterectomy with bladder cuff removel was performed, and histopathological evolution showed a Fuhrman grade 3 clear cell type RCC with low-grade TCC of the pelvis.

## 1. Introduction 

Simultaneous occurrence of renal cell carcinoma (RCC) and transitional cell carcinoma (TCC) in the ipsilateral kidney is a rare entity. There are only about 50 cases reported in the literature to date [1–3].

 Herein, we reported a 73-year-old man who admitted to our clinic with simultaneous RCC and TCC of the left kidney.

## 2. Case Presentation

**Figure 1:**
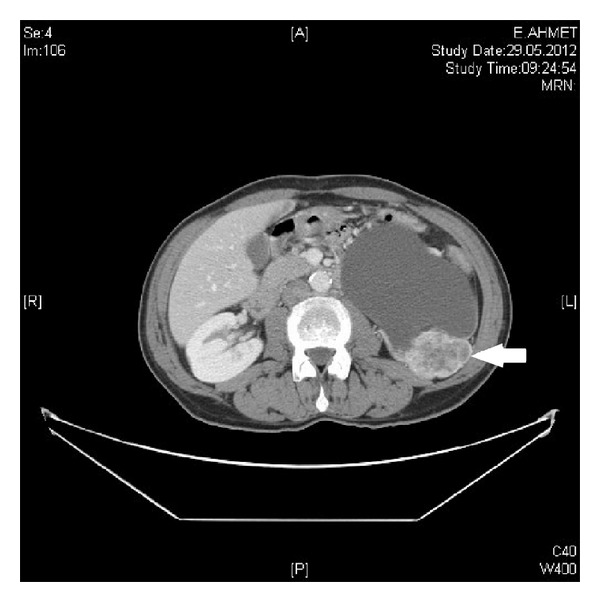
CT image of the solid renal parenchymal mass in the left kidney.

**Figure 2:**
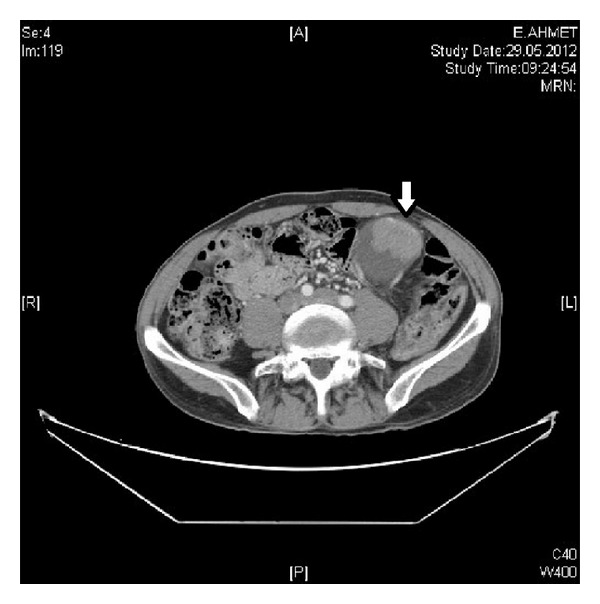
CT image of the solid mass in the left ureteropelvic junction with severe hydronephrosis.

**Figure 3:**
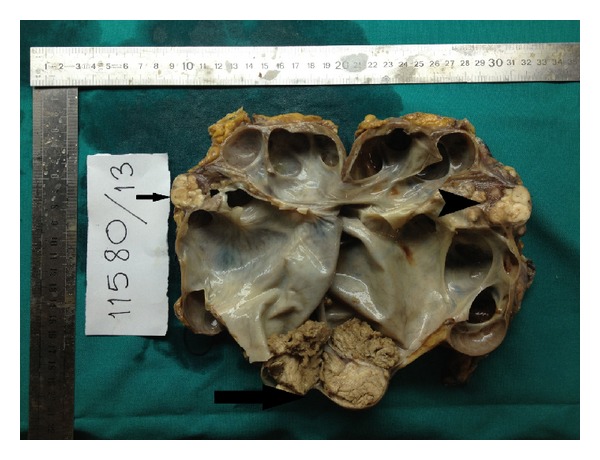
Macroscopic view of RCC (small arrow) and TCC (large arrow) with severe hydronephrosis.

**Figure 4:**
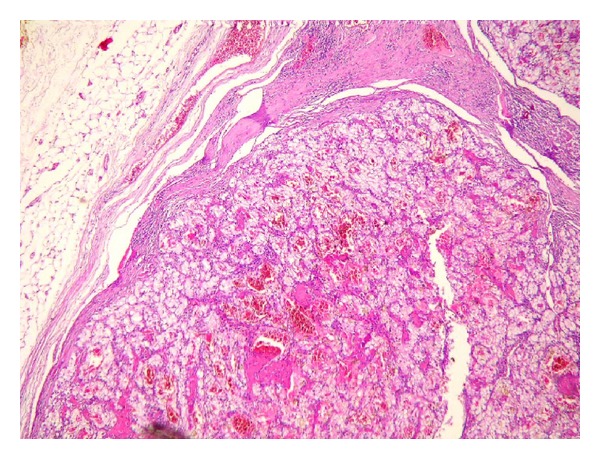
Microscopic overview of the RCC (H-E, ×30).

**Figure 5:**
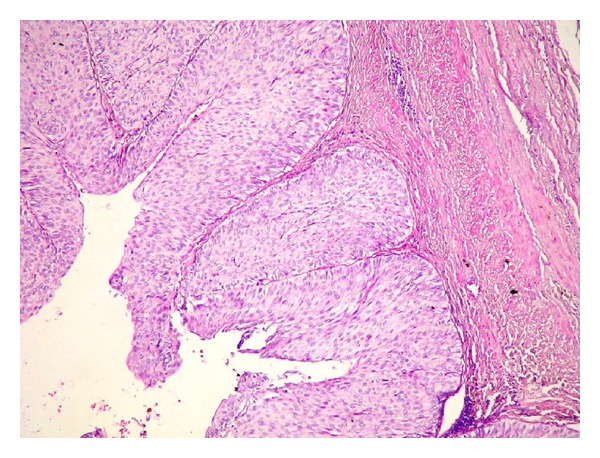
Papillary urothelial carcinoma (H-E, ×30).

 A 73-year-old man who he had suffered from with left flank pain and hematuria was admitted to our clinic. Physical examination and laboratory findings were normal. Patient has a history of ischemic heart disease and 1 pack of cigarette smoking for 40 years. The USG showed grade 4 hydronephrosis and a solid mass with 5 cm diameter in the left kidney. Computed tomography revealed several hydronephrosis and a solid mass with 52 × 41 mm diameters in the middle part of the left kidney. Additionally, a 50 × 45 × 38 mm solid mass was detected at the ureteropelvic junction (UPJ) of the same kidney with normal contralateral kidney (Figures 1 and 2). There was no evidence of metastasis. Cystoscopy revealed no pathological findings, and subsequently left nephroureterectomy with lymphadenectomy was performed. Macroscopic evaluation of the specimen showed severe hydronephrotic left kidney with thin parenchyma and a solid mass with 70 × 70 × 5.5 mm diameters located in the middle part of the kidney without capsular penetration. In addition, a 60 × 50 × 40 mm diameters solid mass with papillomatous components was detected at the ureteropelvic junction (Figure 3). Microscopically, parenchymal mass was detected as a Fuhrman 3 clear cell type RCC, and papillosolid mass at the UPJ was detected as a noninvasive low-grade papillary urothelial carcinoma (Figures 4 and 5). Surgical margins were negative for both tumors. Postoperative 5th day patient was discharged without any complication, and no problems occurred during follow-up period.

## 3. Discussion

 RCC is the commonest solid lesion of the kidney and accounts for approximately 90% of all kidney malignancies [4]. Conversely, primary transitional cell carcinoma (TCC) of the renal pelvis or ureter is a relatively rare disease, and it accounts for less than 1% of genitourinary neoplasms and 5–7% of all urinary tract tumours [5]. Synchronous ipsilateral TCC of the renal pelvis and RCC rarely have been reported in the literature. 

 Several possible aetiological factors have been implicated for primary renal pelvic neoplasms. Although the etiology of coexistence of different type renal neoplasms is still unclear, chronic irritation, hydronephrosis, and urinary calculi have been the most commonly discussed etiologic factors [6]. 

The symptoms of the synchronous RCC and TCC are similar to the solitary RCC or TCC of the kidney. The most common symptom at presentation was haematuria which was seen in 90% of the cases [7, 8]. The mean age at presentation was 65, and male/female ratio was 2/1. The tumors were commonly located on the left kidney [9, 10]. The standard treatment of RCC is the radical nephrectomy or partial nephrectomy for thus small renal carcinomas. However, recurrence rate in the ipsilateral ureteral stump is stated as 30–7% for TCC of the kidney, and high grade recurrences in ureteral stump are associated with poor prognosis [11]. Because of  that, in such cases with synchronous TCC and RCC of the same kidney, ureterectomy with partial cystectomy should be added to the treatment. Furthermore, synchronous or metachronous bladder TCC due to seeding of the tumor cells may occur approximately in 45% of upper urinary tract TCCs [12]. Therefore, cystoscopic evaluation of the bladder should be performed preoperatively. 

 Although synchronous RCC and TCC of the same kidney are a rare condition and there is no certain opinion about the treatment, radical nephroureterectomy with bladder cuff removal may be curative, especially in low-grade tumors.
